# Comparison of diagnostic tests for detecting bovine brucellosis in animals vaccinated with S19 and RB51 strain vaccines

**DOI:** 10.14202/vetworld.2023.2080-2085

**Published:** 2023-10-14

**Authors:** Marcelo Ibarra, Martin Campos, Benavides Hernán, Anthony Loor-Giler, Andrea Chamorro, Luis Nuñez

**Affiliations:** 1 Facultad de Industrias Agropecuarias y Ciencias Ambientales, Carrera Agropecuaria, Universidad Politécnica Estatal del Carchi, Antisana S/N y Av Universitaria, Tulcán Ecuador 040102; 2 Facultad de Ciencias Veterinarias, Universidad Nacional de Rosario, Boulevard Ovidio Lagos y Ruta 33 Casilda-Santa Fe-Argentina; 3 Facultad de Ingeniería y Ciencias Aplicadas, Carrera de Ingeniería en Biotecnología, Universidad de Las Américas, Antigua Vía a Nayón S/N, Quito EC 170124 Ecuador; 4 Facultad de Industrias Agropecuarias y Ciencias Ambientales, Carrera de Enfermeria, Universidad Politécnica Estatal del Carchi, Antisana S/N y Av Universitaria, Tulcán Ecuador 040102; 5 Facultad de Ciencias de la Salud, Carrera de Medicina Veterinaria, Universidad de Las Américas, Antigua Vía a Nayón S/N, Quito EC 170124 Ecuador; 6 One Health Research Group, Universidad de Las Américas, Quito, Ecuador

**Keywords:** agglutination, bovine, brucellosis, vaccination

## Abstract

**Background and Aim:**

The diagnosis of bovine brucellosis in animals vaccinated with strain-19 (S19) and Rose Bengal (RB)-51 strain vaccines can be misinterpreted due to false positives. This study aimed to compare diagnostic tests for detecting bovine brucellosis in animals vaccinated with S19 and RB51 vaccine strains.

**Materials and Methods:**

Two groups of 12 crossbred Holstein calves between 6 and 8 months of age were used. On day 0, blood samples were collected from the animals, and the competitive enzyme-linked immunosorbent assay was used for serological diagnosis of bovine Brucellosis. All animals tested negative. After the first blood collection, the animals were subcutaneously vaccinated: one group received the S19 vaccine and the other received the RB51 vaccine. From the 3^rd^ month after vaccination, all animals were sampled. Sampling was repeated every 2 months until the 7^th^ month. Serological diagnosis of bovine brucellosis was performed using RB, tube serum agglutination test (SAT), SAT with 2-mercaptoethanol (SAT-2Me), and fluorescence polarization assay (FPA).

**Results:**

Animals vaccinated with S19 showed positive results with the RB, SAT, and SAT-2Me tests in all months of post-vaccination diagnosis. In animals vaccinated with S19, FPA showed positive results at months 3 and 5 and negative results at month 7, indicating that this test discriminates vaccinated animals from infected animals 7 months after vaccination. Rose Bengal, SAT, SAT-2Me, and FPA tests showed negative results in animals vaccinated with RB51 in all months of diagnosis.

**Conclusion:**

Animals vaccinated with S19 may test positive for brucellosis using RB, SAT, or SAT-2Me tests 7 months later. Fluorescence polarization assay is an optimal alternative for diagnosing animals in the field, thereby preventing false positives, and consequently, unnecessary confiscations of animals. Animals vaccinated with RB51 tested negative with RB, SAT, SAT-2Me, and FPA tests in all months of diagnosis, confirming that the tests are ineffective for diagnosing brucellosis caused by rough strains.

## Introduction

Bovine brucellosis is a contagious disease caused by *Brucella* spp. (phylum: α-2 Proteobacteria), with worldwide distribution, and reproductive conditions that affect both males and females [1]. *Brucella* causes productive and reproductive loss in livestock. Notably, in dairies, *Brucella* causes abortion, accompanied by retained placenta, birth of weak calves, low milk production in females [2, 3], and epididymitis and orchitis in males [4].

Bovine brucellosis has been eradicated from many parts of the world, especially North America and Western Europe, but remains endemic to certain areas, particularly in Asia, Africa, and Latin America [5], where key control strategies include mass vaccination of animals at risk, with serological diagnosis. The *Brucella abortus* strain-19 (S19) vaccine was developed in 1923 from a natural attenuation [6] and has been used for ~50 years. However, it presents drawbacks such as the inference in conventional diagnostic tests, the non-possibility of vaccination of adult animals, and the risks to the veterinarians [1]. A mutant strain of *B*. *abortus* strain 2308 Rose Bengal (RB)-51 was isolated in 1982 from *B. abortus* biovar 1 [7], to generate a vaccine that can be used for bovines of all ages and does not infer in the conventional serological diagnosis [8]. Several studies have shown that the S19 and RB51 vaccines provide 65%–75% protection against infection [7–9].

The World Organization for Animal Health (WOAH) recommends using multiple serological tests for diagnosing brucellosis to overcome individual limitations of each test. Performing multiple serological tests increases sensitivity and reduces percentage of false negatives. Due to these drawbacks, population screening in control programs requires the use of combined serological tests [10]. Although conventional serological tests are key in bovine brucellosis control and eradication programs, they cannot discriminate between naturally infected and S19-vaccinated animals, because they detect antibodies produced against the O chain of lipopolysaccharide (LPS-O) on the membrane of *Brucella* spp., which is present in both field strains and vaccines [11]. This has led to the development of several diagnostic tests for bovine brucellosis, including agglutination, enzymatic, and cellular immunity tests, but none is considered a gold standard. The characteristics of tests used in eradication plans worldwide are being investigated, because they generate false-positive and false-negative outcomes [12]. The most common screening tests for diagnosing brucellosis include: The RB test, which is a qualitative agglutination test that can be rapidly observed; the tube serum agglutination test (SAT); and SAT with 2-mercaptoethanol (SAT-2Me). Rapid tests specifically detect antibodies against *Brucella* spp. LPS [13]. Competitive enzyme-linked immunosorbent assay (cELISA) is used as a confirmatory serological test because of its high specificity to distinguish antibodies produced in response to a vaccine or natural infection [14]. This test uses the LPS-O-specific monoclonal antibody M-84, and the antigen–antibody reaction is detected that is quantified through enzymatic meters [13]. However, due to its high specificity, the fluorescence polarization assay (FPA) can be considered a confirmatory test for bovine brucellosis [15]. Although available *B. abortus* vaccines are effective against brucellosis, they have a number of drawbacks, including interference with diagnostic tests, pathogenicity to humans, and potential to cause abortions in expectant animals. Due to the presence of LPS in the S19 vaccine, vaccination of animals with this strain induces an immune response against LPS-O that is strikingly similar to that induced by natural infection. Therefore, distinguishing between vaccinated and infected animals is impossible [16]. In contrast, vaccination with *B. abortus* strain RB51 did not induce antibodies detected by the conventional assays used to diagnose brucellosis. In addition, there is no commercially available test to detect RB51- or S19-vaccinated animals, which would be beneficial for evaluating vaccination programs [17]. Bovine brucellosis is endemic to Ecuador, which has a bovine brucellosis control and eradication plan based on epidemiological surveillance, serological diagnosis, slaughter of seropositive animals, vaccination, and training. Vaccination is one of the fundamental pillars of disease eradication; however, it is performed without the care required by government entities. Vaccine strains S19 and RB51 are used without control. In certain cases, the two strains have been used in the same animal, without an adequate cold chain and sanitary records [10]. Therefore, the diagnostic tests used in Ecuador cannot identify brucellosis-free farms due to interference from false-positive and negative results [18–20].

This study aimed to compare diagnostic tests for detecting bovine brucellosis in animals vaccinated with S19 and RB51 vaccine strains.

## Materials and Methods

### Ethical approval

All procedures conducted in the present study were approved by the Committee on the Care and Use of Laboratory and Domestic Animal resources of the Agency of Regulation and Control of Phytosanitary and Animal Health of Ecuador (AGROCALIDAD), under the approval serial number #INT/DA/019.

### Study period and location

The study was conducted from February to November 2022 at a farm in San Vicente, El Carmelo parish, Tulcan Canton, Carchi Province, Ecuador.

### Experimental design

Two groups of total 12 (six in each group) crossbred Holstein calves between 6 and 8 months of age were used. The animals were kept in paddocks with forage and water *ad libitum*. Blood samples were taken from the coccygeal vein in sterile tubes without anticoagulant (BD, New York, United States), to obtain serum for the serological diagnosis of *Brucella* spp., before and after vaccination. Serological diagnosis (day 0) was made using the cELISA test (Svanova by Indical Bioscience, Uppsala, Sweden), which is considered a confirmatory test in Ecuador for the diagnosis of bovine brucellosis, according to resolution No. 025 Art. 8 (AGROCALIDAD, Regulation and Control Agency for Plant and Animal Health. On day 1, six animals each were vaccinated subcutaneously with a single dose of S19 (5–8 × 10^10^ colony-forming unit [CFU]) and RB51 (1.6 × 10^10^ CFU) [10].

In the 3^rd^ month after vaccination, blood samples (10 mL) were taken to obtain serum from all animals. This procedure was repeated every 2 months until the 7^th^ month. The samples were serologically tested for antibodies against *Brucella* spp. using RB, SAT, SAT-2Me, and FPA, at the veterinary diagnostic laboratory of the State Polytechnic University of Carchi.

### Serological tests

Competitive enzyme-linked immunosorbent assay was performed in duplicate using Svanovir *Brucella*-Ab C-ELISA (Svanova Biotech AB). Percent inhibition was calculated using the following formula:

Subtracting 100 for the division of the average optical densities (OD) of the samples with the OD of the conjugate cutoff ≥30% inhibition was considered positive and <30% inhibition was considered negative.

### Rose Bengal

**Figure-1:**
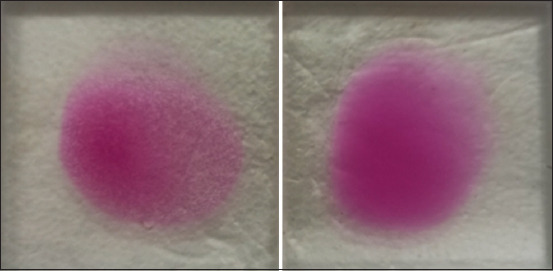
Rose Bengal test-Positive (left); Negative (right).

The RB test was performed according to the protocol established by the OIE. Rose Bengal Brucellosis Antigen (Idexx, Hoofddorp, The Netherlands), which is a bacterial suspension of *Brucella* stained with RB, was used as the antigen. The antigen and sera were placed at room temperature (23°C) for 60 min before use. Next, 30 μL of serum was placed on a glass plate and mixed with 30 μL of the antigen. The plate was homogenized for 4 min at 23°C. The presence or absence of agglutination was considered positive or negative, respectively (Figure-1).

### Serum agglutination test and SAT-2Me

**Figure-2:**
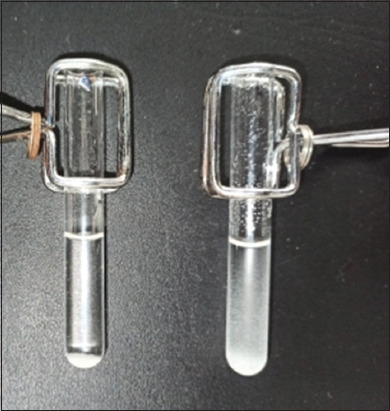
Sero Agglutination Tube with two Mercaptoethanol test – Positive (left); Negative (right).

Serum agglutination test and SAT-2Me used a 4.5% suspension of *B*. *abortus* 1119-3 as antigen, as well as 0.5% phenolated saline and 0.1 M 2Me as diluents, respectively. The protocol followed was proposed in 2009 by the WOAH [21]. Considerations for the interpretation of results include: Complete agglutination is the degree of agglutination in each test, where the liquid of the serum-antigen mixture appears transparent and translucent and gentle agitation does not disperse the aggregates. For incomplete agglutination, the serum-antigen mixture is partially cloudy and moderate shaking fails to disperse the clumps. Negative agglutination occurs when the serum-antigen mixture appears cloudy and moderate shaking does not reveal lumps. Reading and interpreting results of the slow agglutination test in a tube in the presence of “SAT-2Me” must be performed according to the same criteria as the slow agglutination test in a tube (“SAT”) (Figure-2).

### Fluorescence polarization assay

Fluorescence polarization assay was performed according to the specifications of *Brucella* Antibody Test Kit FPA (EllieLab, Milwaukee, United States). The FPA kit uses fluorescein-conjugated O-polysaccharide from *B*. *abortus*. The sera and controls (20 μL) were placed inside borosilicate tubes with the diluent provided by the kit (1 mL) and incubated for 3 min at 23°C, to make blank reading of all the samples and controls. Then, 10 μL of the antigen was incubated with fluorescein for 3 min at 23°C. Milli-polarization (mP) values of all samples and controls were obtained. Cutoff ≥ 89.9 mP indicated a positive result [10].

### Statistical analysis

The results were analyzed using descriptive statistics in R program 4.3.1 version (R Foundation for Statistical Computing, Vienna, Austria).

## Results

Serological diagnosis revealed that on day 0, the animals did not present antibodies against *Brucella* spp. The animals vaccinated with S19 showed the presence of antibodies (positive diagnosis) for *Brucella* spp. using SAT in all collection periods (months 3, 5, and 7). Rose Bengal and SAT-2Me presented a positive diagnosis in months 3 and 5 in all animals; however, in month 7, positive results were observed in five animals and negative results in one animal. In months 3 and 5, FPA revealed a positive diagnosis in all animals, but by month 7, all animals had a negative diagnosis (Table-1). The animals that received the RB51 vaccine did not exhibit any positive results during any of the sampling periods or diagnostic procedures.

**Table-1:** Determination of antibodies to *Brucella* spp. by screening agglutination tests.

Vaccine	Animal	Detection of antibodies for *Brucella*spp. at different post-vaccination times											

		Month 3				Month 5				Month 7			
		
		RB	SAT	SAT-2Me	FPA	RB	SAT	SAT-2Me	FPA	RB	SAT	SAT-2Me	FPA
S19	1	+	+	+	+	+	+	+	+	+	+	+	-
	2	+	+	+	+	+	+	+	+	+	+	+	-
	3	+	+	+	+	+	+	+	+	-	+	-	-
	4	+	+	+	+	+	+	+	+	+	+	+	-
	5	+	+	+	+	+	+	+	+	+	+	+	-
	6	+	+	+	+	+	+	+	+	+	+	+	-
RB51	7	-	-	-	-	-	-	-	-	-	-	-	-
	8	-	-	-	-	-	-	-	-	-	-	-	-
	9	-	-	-	-	-	-	-	-	-	-	-	-
	10	-	-	-	-	-	-	-	-	-	-	-	-
	11	-	-	-	-	-	-	-	-	-	-	-	-
	12	-	-	-	-	-	-	-	-	-	-	-	-

RB=Rose Bengal test, SAT=Sero agglutination tube, SAT-2Me=Sero agglutination tube with 2 Mercaptoethanol, FPA=Fluorescence polarization assay

## Discussion

Our study demonstrates that unlike the screening tests (RB, SAT, and SAT-2Me) used for the serological detection of *Brucella* spp., FPA has great potential for detecting animals that are not infected with *Brucella* spp., because it can distinguish between antibodies produced in infected and vaccinated animals. This is due to the test’s principle, in which all the molecules in solution rotate randomly. The size of the molecules determines the rotation range, which refers to the formation of immune complexes between antigen and immunoglobulin (Ig)G-type antibodies. In periods where the concentration of IgM begins to decrease, negative results are observed in animals experimentally vaccinated with S19 at 7 month post-vaccination. In contrast, screening tests, being general agglutination tests, continue to identify antibodies generated in response to vaccines several months post-vaccination [22–24].

Moreover, SAT is a highly sensitive test that allows detection of IgM-type antibodies, which are the first to appear after an infection. However, SAT presents problems of low specificity due to non-specific antigen–antibody reactions. Serum agglutination test variants (SAT-Rivanol, SAT-EDTA, and SAT-2Me) use acidified antigens, allowing for the formation of immunocomplexes between antigens and IgG-type antibodies. Similarly, the RB test (at pH 3.65) allows detection of IgG-type antibodies, reducing non-specific binding associated with IgM-type antibodies [15].

Similar results have been obtained with RB, SAT, and SAT-2Me because they use the same principle of agglutination on slides and the antigen uses a suspension of *B*. *abortus* [25]. According to Aparicio Bahena *et al*. [26], routine or screening tests, such as RB, SAT, buffered plate antigen, and milk ring test, have high diagnostic sensitivity; however, their specificity is low when differentiating vaccinated from infected animals [26].

Positive results from RB, SAT, and SAT-2Me tests can be considered false positives due to the low specificity of these tests, which is caused by cross-reactions with other LPS-O-containing bacteria. Similar results were described by Nielsen *et al*. [27], where the bacteria causing this cross-reaction included *Escherichia hermanni* and *Escherichia coli* O157, as well as *Salmonella* O:30 and *Stenotrophomonas maltophilia*. According to Ron-Román *et al*. [25], vaccination of cattle with S19 produces agglutinating antibodies that interfere with diagnostic tests based on the principle of agglutination.

In animals vaccinated with S19, FPA presented positive results for up to 5 months of sampling. This diagnosis should be considered a false-positive because it is attributable to cross-reactions due to S19 vaccination, where after vaccination with strains with epitopes similar to the causal agent, very high levels of antibodies are produced, especially IgM and IgG [28].

Because FPA has high specificity in animals vaccinated with S19 [11, 21, 29], it is possible to observe negative results for FPA beginning in the 7^th^ month, where the specificity of FPA, and consequently, its ability to distinguish vaccinated from naturally infected animals are 98.60% and 99.80%, respectively, as long as the estimated duration is 6–7-month post-vaccination at the time of the test.

Animals vaccinated with the RB51 strain presented negative results in all the diagnostic tests under study (RB, SAT, SAT-2Me, and FPA) and in all the sampling months (Table-1).

The negative results of the RB, SAT, SAT-2Me, and FPA diagnostic tests are attributed to the use of suspensions of *B*. *abortus* as antigen, which is a smooth bacterium, due to the presence of LPS-O, Vargas [30], whereas the vaccine applied in this group of animals, which was RB51, is a rough mutant strain lacking the side chain “O” of the LPS, which induces the production of other types of antibodies, in which the antigen–antibody ratio of the diagnostic tests under study is null, as mentioned by Schurig *et al*. [12] and Cheville *et al*. [31].

Two types of commercial vaccines are available worldwide for bovine brucellosis: S19 and RB51, which are live vaccines, with similar degrees of immunity; however, they sometimes trigger the production of agglutinating antibodies that interfere with all serological diagnostic tests [32, 33]. In the case of the RB, SAT, and SAT-2Me diagnostic tests performed on animals vaccinated with S19, positive results were obtained up to the 7^th^ month of sampling, whereas for FPA, negative results were seen at 7^th^ month of diagnosis. Thus, FPA can differentiate vaccinated animals, after the highest peak of vaccine immunity passes in 6- and 7-month post-vaccination. A comparison of the diagnostic capacity of FPA with cELISA (test recognized by AGROCALIDAD) revealed a high diagnostic correlation [9].

In animals vaccinated with RB51, the RB, SAT, SAT-2Me, and FPA tests showed negative results in all months of diagnosis, proving that the antibodies induced by RB51 cannot be detected (there is no antigen–antibody interaction) by the diagnostic screening tests for bovine brucellosis [34].

Thus, in cattle herds where the RB51 vaccine strain is used correctly, the use of diagnostic screening tests, such as RB, could be considered unequivocally. In addition, because the RB51 strain is a rough strain, its immunological efficiency cannot be determined with traditional diagnostic tests, because these tests use antigens obtained from smooth strains. Therefore, antigens from rough strains must be used to evaluate the immunological efficiency of RB51.

## Conclusion

In this study, FPA was shown to be a useful tool for the rapid and effective detection of antibodies against *Brucella* spp. in cattle, contributing significantly to the differentiation between animals that are infected and those that appear healthy or are vaccinated (7 months after vaccination). However, FPA is not useful for detecting antibodies produced by rough strains. Therefore, FPA is the most recommended test in Ecuador and other countries for detecting animals infected with *Brucella* spp.

## Authors’ Contributions

MI and MC: Sample and data collection. MI, MC, BH, AL, AC, and LN: Study design, drafted the manuscript, laboratory work, and data analysis. All authors have read, reviewed, and approved the final manuscript.

## References

[1] Olsen S, Tatum F (2010). Bovine brucellosis. Vet. Clin. N. Am. Food Anim. Pract.

[2] Yamamoto T, Tsutsui T, Nishiguchi A, Kobayashi S (2008). Evaluation of surveillance strategies for bovine brucellosis in Japan using a simulation model. Prev. Vet. Med.

[3] England T, Kelly L, Jones R.D, MacMillan A, Wooldridge M (2004). A simulation model of brucellosis spread in British cattle under several testing regimes. Prev. Vet. Med.

[4] Etefa M, Kabeta T, Merga D, Debelo M (2022). Cross-sectional study of seroprevalence and associated risk factors of bovine brucellosis in selected districts of Jimma Zone, South Western Oromia, Ethiopia. Biomed. Res. Int.

[5] Seleem M.N, Boyle S.M, Sriranganathan N (2010). Brucellosis:A re-emerging zoonosis. Vet. Microbiol.

[6] Wang S, Wang W, Sun K, Bateer H, Zhao X (2020). Comparative genomic analysis between newly sequenced *Brucella abortus* vaccine strain A19 and another *Brucella abortus* vaccine S19. Genomics.

[7] Olsen S.C, Stoffregen W.S (2005). Essential role of vaccines in brucellosis control and eradication programs for livestock. Expert Rev. Vaccines.

[8] Abnaroodheleh F, Emadi A, Dashtipour S, Jamil T, Mousavi Khaneghah A, Dadar M (2023). Shedding rate of *Brucella* spp. in the milk of seropositive and seronegative dairy cattle. Heliyon.

[9] de Oliveira M.M, Pereira C.R, de Oliveira I.R.C, Godfroid J, Lage A.P, Dorneles E.M.S (2022). Efficacy of *Brucella*
*abortus* S19 and RB51 vaccine strains:A systematic review and meta-analysis. Transbound. Emerg. Dis.

[10] Paucar V, Ron-Román J, Benítez-Ortiz W, Celi M, Berkvens D, Saegerman C, Ron-Garrido L (2021). Bayesian estimation of the prevalence and test characteristics (Sensitivity and specificity) of two serological tests (RB and SAT-EDTA) for the diagnosis of bovine brucellosis in small and medium cattle holders in Ecuador. Microorganisms.

[11] Chacón-Díaz C, Zabalza-Baranguá A, Román B.S, Blasco J.M, Iriarte M, Salas-Alfaro D, Hernández-Mora G, Barquero-Calvo E, Guzmán-Verri C, Chaves-Olarte E, Grilló M.J, Moreno E (2021). *Brucella abortus* S19 GFP-tagged vaccine allows the serological identification of vaccinated cattle. PLoS One.

[12] Schurig G.G, Roop R.M, Bagchi T, Boyle S, Buhrman D, Sriranganathan N (1991). Biological properties of RB51;a stable rough strain of *Brucella abortus*. Vet. Microbiol.

[13] Khurana S.K, Sehrawat A, Tiwari R, Prasad M, Gulati B, Shabbir M.Z, Chhabra R, Karthik K, Patel S.K, Pathak M, Iqbal Yatoo M, Gupta V.K, Dhama K, Sah R, Chaicumpa W (2021). Bovine brucellosis-a comprehensive review. Vet. Q.

[14] Shurbe M, Wondimu A, Eshetu N, Seyoum W, Tora E, Simeon B, Rufael T, Sombo M (2023). Detection of antibodies against brucellosis and associated risk factors in cross breed dairy cattle in smallholder farmers, Southern Ethiopia. Vet. Med.(Auckl).

[15] Nielsen K (2002). Diagnosis of brucellosis by serology. Vet. Microbiol.

[16] Tahmo N.B, Wirsiy F.S, Nnamdi D.B, Tongo M.P, Lawler J.V, Broadhurst M.J, Wondji C.S, Brett-Major D.M (2022). An epidemiological synthesis of emerging and re-emerging zoonotic disease threats in Cameroon, 2000–2022:A systematic review. IJID Reg.

[17] Faria A.R, Dorneles E.M.S, Pires S.D.F, de Andrade H.M, Lage A.P (2020). Immunoproteomics of *Brucella*
*abortus* reveals potential of recombinant antigens for discriminating vaccinated from naturally infected cattle. Microb. Pathog.

[18] Bardenstein S, Grupel D, Blum S.E, Motro Y, Moran-Gilad J (2023). Public and animal health risks associated with spillover of *Brucella*
*melitensis* into dairy farms. Microb. Genom.

[19] Warioba J.P, Karimuribo E.D, Komba E.V.G, Kabululu M.L, Minga G.A, Nonga H.E (2023). Occurrence and risk factors of brucellosis in commercial cattle farms from selected districts of the Eastern Coast Zone, Tanzania. Vet. Med. Int.

[20] Samartino L.E, Fort M, Gregoret R, Schurig G.G (2000). Use of *Brucella abortus* vaccine strain RB51 in pregnant cows after calfhood vaccination with strain-19 in Argentina. Prev. Vet. Med.

[21] Ramírez C, Ernst S, Elvinger F (2009). Serological response to brucellosis vaccination in bovines from a free herd vaccinated with two doses of RB51. Arch. Med. Vet.

[22] Gall D, Nielsen K, Bermudez M.R, Moreno F, Smith P (2002). Fluorescence polarization assay for detection of *Brucella*
*abortus* antibodies in bulk tank bovine milk samples. Clin. Diagn. Lab. Immunol.

[23] Mengele I.J, Shirima G.M, Bwatota S.F, Motto S.K, de Clare Bronsvoort B.M, Komwihangilo D.M, Lyatuu E, Cook E.A.J, Hernandez-Castro L.E (2023). The Status and risk factors of brucellosis in smallholder dairy cattle in selected regions of Tanzania. Vet. Sci.

[24] Tulu D (2022). Bovine brucellosis:Epidemiology, public health implications, and status of brucellosis in Ethiopia. Vet. Med. (Auckl).

[25] Ron-Román J, Ron-Garrido L, Abatih E, Celi-Erazo M, Vizcaíno-Ordóñez L, Calva-Pacheco J, González-Andrade P, Berkvens D, Benítez-Ortíz W, Brandt J, Fretin D, Saegerman C (2019). Bayesian evaluation of three serological tests for detecting antibodies against *Brucella* spp. Among humans in the Northwestern Part of Ecuador. Am. J. Trop. Med. Hyg.

[26] Aparicio Bahena A, Aparicio E.D, Andrade L.H, González R.P, Silva E.A, Güemes F.S (2003). Serological and bacteriological evaluation of a bovine herd infected with brucellosis and revaccinated with a reduced dose of *Brucella abortus* cepa 19. Técnica pecuaria en méxico. Téc. Pecu Méx.

[27] Nielsen K, Smith P, Widdison J, Gall D, Kelly L, Kelly W, Nicoletti P (2004). Serological relationship between cattle exposed to *Brucella*
*abortus*, *Yersinia enterocolitica* O:9 and *Escherichia coli* O157:H7. Vet. Microbiol.

[28] Nielsen K, Gall D, Jolley M, Leishman G, Balsevicius S, Smith P, Nicoletti P, Thomas F (1996). A homogeneous fluorescence polarization assay for detection of antibody to *Brucella abortus*. J. Immunol. Methods.

[29] Dabral N, Burcham G.N, Jain-Gupta N, Sriranganathan N, Vemulapalli R (2019). Overexpression of wbkF gene in *Brucella*
*abortus* RB51WboA leads to increased O-polysaccharide expression and enhanced vaccine efficacy against *B. abortus* 2308, *B. melitensis* 16M, and B. SUIS 1330 in a murine brucellosis model. PLoS One.

[30] Francisco J, Vargas O (2002). Brucellosis in Venezuela. Vet. Microbiol.

[31] Cheville N.F, Olsen S.C, Jensen A.E (1996). Effects of age at vaccination on efficacy of *Brucella*
*abortus* strain RB51 to protect cattle against brucellosis. Am. J. Vet. Res.

[32] Saidu A.S, Singh M, Kumar A, Mahajan N.K, Mittal D, Chhabra R, Joshi V.G, Musallam I.I, Sadiq U (2022). Studies on intra-ocular vaccination of adult cattle with reduced dose *Brucella*
*abortus* strain-19 vaccine. Heliyon.

[33] Holt H.R, Walker M, Beauvais W, Kaur P, Bedi J.S, Mangtani P, Sharma N.S, Gill J.P.S, Godfroid J, McGiven J, Guitian J (2023). Modelling the control of bovine brucellosis in India. J. R. Soc. Interface.

[34] Wakjira B.S, Jorga E, Lakew M, Olani A, Tadesse B, Tuli G, Belaineh R, Abera S, Kinfe G, Gebre S (2022). Animal Brucellosis:Seropositivity rates, isolation, and molecular detection in southern and central Ethiopia. Vet. Med. (Auckl).

